# Lateral modulation of orientation perception in center-surround sinusoidal stimuli: Divisive inhibition in perceptual filling-in

**DOI:** 10.1167/jov.20.9.5

**Published:** 2020-09-04

**Authors:** Yih-Shiuan Lin, Chien-Chung Chen, Mark W. Greenlee

**Affiliations:** Institute of Experimental Psychology, University of Regensburg, Regensburg, Germany; Department of Psychology, National Taiwan University, Taipei, Taiwan; Neurobiology and Cognitive Science Center, National Taiwan University, Taipei, Taiwan

**Keywords:** surround modulation, perceptual filling-in, lateral inhibition, orientation selectivity, spatial vision

## Abstract

The perception of a target stimulus may be altered by its context. Perceptual filling-in is thought to be one example of lateral modulation, in which the percept of a central blank area is replaced by that of the surround. We investigated the mechanisms in eccentric vision underlying filling-in by selectively adapting the center (pedestal adapter), surround (annulus adapter), or both (disk adapter) in a sinusoidal grating and observed how the adaptation influences the orientation percept of a subsequently presented Gabor target, located at the same position as the adapter center. In a binary choice task, observers were to judge the orientation (clockwise or counterclockwise) of the target after adaptation. The tilt aftereffect (TAE), corresponding to an illusory tilt of a physically vertical Gabor target, depended both on the adapter orientation and the adapter type. The TAE, peaked between 10 degrees and 20 degrees adapter orientation, was strongest in the pedestal, followed by the disk, and weakest in the annulus adapter conditions. The difference between the disk and pedestal conditions implies lateral inhibition from the surround. Lacking physical overlap with the target, the annulus adapter nonetheless induced a small but significant TAE in the central area. The effect of filling-in on the TAE was estimated by comparing the results from trials with and without subjectively reported filling-in during adaptation to the annulus adapter. The TAE was greater when filling-in occurred during adaptation, suggesting a stronger lateral modulation effect on trials where filling-in was induced. The data were fit by a variant of a divisive inhibition model, in which the adaptation effect is captured by the increase of an additive constant in the denominator of the response function, whereas the surround modulation in the adapter is modeled by an excitatory sensitivity in the numerator.

## Introduction

The effect of context on a centrally located target stimulus has long been recognized in visual sciences (Gilbert & Wiesel, 1990; Pessoa & De Weerd, 2003). At the retinal level, horizontal cells (HCs) carry out the first lateral projection in the retina (Baylor, Fuortes, & O'Bryan, 1971). Every HC receives synaptic signals from several photoreceptors and the former transmits feedback signals back to the photoreceptors to alter their neurotransmitter release. This HC feedback is mostly inhibitory. A group of photoreceptors, which send synaptic signals directly to a bipolar cell, forms the receptive field center of that bipolar cell. Meanwhile, HCs collect signals from a larger group of photoreceptors surrounding the centrally located photoreceptors and these form the receptive field surround. When the surround photoreceptors are activated, they send excitatory signals to the HCs, the HCs in turn send inhibitory feedback to the centrally located photoreceptors, mediating the response of the bipolar cell. This creates the typical center-surround/excitatory-inhibitory receptive fields (RFs) often observed in bipolar cells. This simple circular-symmetric shape of RFs can be found up in the retinal ganglion cells and neurons in the lateral geniculate nucleus (LGN). Owing to their symmetrical shape, these RFs are nonselective to stimulus orientation. In the primary visual cortex, or V1, RFs become more selective to the orientation, motion direction, contrast, and size of the stimulus (Hubel & Wiesel, 1962). From here onward, lateral interactions between regions in the visual field form a complex network with both feedforward and feedback circuits.

At the cortical level, lateral modulation occurs also beyond the center-surround classical receptive field (CRF). One example is the surround suppression observed when a neuron's receptive field is stimulated with an oriented grating stimulus. The surround stimuli themselves do not induce any neural activity in the recorded neuron, but can nevertheless suppress (predominantly) or facilitate the CRF response activated by a central stimulus (Barlow, 1953; Cavanaugh, Bair, & Movshon, 2002a, Cavanaugh, Bair, & Movshon, 2002b; Hubel & Wiesel, 1959; Maffei & Fiorentini, 1976). This surround modulation requires long-range integration of information across multiple CRFs. Many studies show that the surrounding area beyond the CRF can have similar orientation and spatial frequency tuning as those of the CRF, suggesting that this form of lateral inhibition is feature-specific (Blakemore & Tobin, 1972; Cavanaugh, Bair, & Movshon, 2002b; DeAngelis, Freeman, & Ohzawa, 1994; Gilbert & Wiesel, 1990; Nelson & Frost, 1978; Ozeki, Sadakane, Akasaki, Naito, Shimegi, & Sato, 2004; Sillito, Grieve, Jones, Cudeiro, & Davls, 1995). Different paradigms have been suggested to study this long-range lateral inhibition. Using the method consisting of an expanding patch or an expanding annulus to study the surround beyond the CRF, various groups discovered two surround areas (near and far surrounds) that contribute different contrast-dependent effects on the CRF (Angelucci & Bressloff, 2006; Ichida, Schwabe, Bressloff, & Angelucci, 2007; Sengpiel, Sen, & Blakemore, 1997; Shushruth, Ichida, Levitt, & Angelucci, 2009).

Another common way to investigate lateral modulation is through a psychophysical lateral masking paradigm, in which target detection threshold is estimated in the presence of surround flankers on either side of the target (Polat & Sagi, 1993, 1994; Zenger & Sagi, 1996). These authors reported that the flanker effect depended on the relative orientation between the flankers and the target. When the target and flankers were collinear, the target detection threshold was significantly reduced. On the other hand, when the flankers were orthogonal to the target, no target facilitation was found. Solomon, Watson, and Morgan (1999) further investigated such collinear facilitation flanker effects by using flankers of the same or opposite-sign contrast as the target and found that the opposite-sign flankers created a much weaker facilitation on the target than the same-sign flankers. Later, the same authors discovered that adding noncollinear flankers surrounding the central target cancelled such facilitation effects (Solomon & Morgan, 2000). Chen and Tyler (2001, 2002) used a dual-mask paradigm to investigate the flanker effect. They added a pedestal, which occupied the same spatial location with the same spatio-temporal properties of the target. Without the flankers, the target detection threshold first decreased (facilitation) then increased (suppression) with the pedestal contrast (i.e. the well-known dipper effect). The collinear flankers reduced the target threshold when no pedestal was presented and increased the target threshold at high pedestal contrast. The orthogonal flankers did not produce the facilitation effect, but instead led to a suppression effect at high pedestal contrast. They concluded that there are two flanker effects, one narrowly and the other broadly tuned to flanker orientation. They proposed a divisive inhibition model to explain the data, suggesting that the observed lateral modulation is subject to a normalization process. In a later functional magnetic resonance imaging (fMRI) experiment, (Chen, 2014) explored the neural correlates corresponding to these two inhibitory components. Two effects were evident in his results: first a general inhibition that reduced the target BOLD signal in the presence of the flankers, and second, a flanker-specific inhibition that was stronger when the flanker shared the target orientation (collinear flanker effect). In another line of work, Meese and colleagues (Meese & Holmes, 2007; Meese, Summers, Holmes, & Wallis, 2007) used either annular or superimposed masks of various sizes to investigate how different cross-oriented surrounds can affect the contrast detection and discrimination thresholds of a Gabor target. They reported cross-orientation facilitation as well as cross-orientation suppression from both types of mask. The observed lateral modulation effect varied with spatial and temporal frequencies and masking area.

It is suggested that perceptual filling-in is a visual phenomenon that results directly from center-surround modulation (Anstis & Greenlee, 2014; Pessoa & De Weerd, 2003; Ramachandran & Gregory, 1991). Filling-in describes the effect when the visual system integrates surround features to compensate for occluded or absent information in the center. Filling-in can be experienced in the blind spot, with retinal scotoma, or for artificial scotoma. The blind spot is the part of the visual field corresponding to a region on the retina where the optic nerve exits the eye, thus lacking photoreceptors to detect variations in light. Humans do not experience a dark hole corresponding to the blind spot (even with monocular viewing) because the visual system interpolates and fills in the blank by extracting information at the edge or surround regions (Ramachandran, 1992). Neurophysiological studies reported active neural response in early visual cortex in the projection zone corresponding to the blind spot (Fiorani Júnior, Rosa, Gattass, & Rocha-Miranda, 1992; Komatsu, Kinoshita, & Murakami, 2000, Komatsu, Kinoshita, & Murakami, 2002; Matsumoto & Komatsu, 2005) or to that evoked by artificial scotoma (De Weerd, Gattass, Desimone, & Ungerleider, 1995; Sasaki & Watanabe, 2004), indicating that the central filled-in regions were modulated by the surround neurons that receive visual input. Such perceptual completion has been observed in the retinal scotoma of macular degeneration patients (Zur & Ullman, 2003). Furthermore, scotoma can be induced artificially with the help of strict central fixation or gaze-contingent displays. Ramachandran and Gregory (1991) placed a gray-homogeneous square (the artificial scotoma) in a twinkling noise background. After prolonged steady fixation, the gray square was filled in by the noise background. Morgan, McEwan, and Solomon (2007) presented a dynamic noise pattern with two artificial scotomata on the left and right visual fields as the adapter. When the field was switched off (after participants reported the scotomata being filled-in) and replaced by a mean luminance background, flickering phantasms were perceived. To estimate the effect of such phantasms, the authors presented two small noise patches in the scotoma regions after adaptation, one containing both the target and the pedestal, and the other only the pedestal. Participants were to choose the patch with the target. The target contrast threshold increased after adaptation. In addition, when the adapting field was presented to only one eye, the target threshold increased on both eyes, suggesting a cortical origin of such an adaptation effect.

The functional role of lateral modulation during filling-in remains unclear. Although some studies reported increased neural activities in the filled-in regions, suggesting that lateral excitation is involved (De Weerd, Gattass, Desimone, & Ungerleider, 1995; Komatsu, 2006; Matsumoto & Komatsu, 2005; Roe, Lu, & Hung, 2005; Sasaki & Watanabe, 2004), others proposed that lateral inhibition from the surround might also play a role. Tong and Engel (2001) conducted a binocular rivalry fMRI experiment and measured the activity of the cortical area corresponding to the eccentric visual field location of the blind spot of one eye. When the stimuli were presented, the BOLD signals increased when the ipsilateral-eye image was dominant and decreased when the blind-spot eye image was dominant. This finding suggested a monocular lateral inhibition in V1 neurons when stimulus was presenting at the blind spot. In a magnetoencephalography (MEG) experiment when a uniformly illuminated target disappeared and was replaced by the dynamic background texture, the MEG power at the target frequency was reduced (Weil, Kilner, Haynes, & Rees, 2007). Such power reduction suggests a decreased neural representation of the invisible (filled-in) stimuli in visual cortex. The same group later applied the same filling-in paradigm in an fMRI study and reported again BOLD signal reduction in the V1 and V2 area representing the filled-in target (Weil, Watkins, & Rees, 2008). These seemingly contradicting results led to the possibility that the underlying mechanism of filling-in could be complex involving multiple processes that include both lateral excitation and inhibition.

Although many groups have investigated the lateral modulation in vision with various paradigms, such as lateral masking and visual crowding, few have studied the lateral modulation by selectively adapting the central and/or surround mechanism and observed the effect on the central percept.

To understand more about lateral modulation in human vision, we designed an adaptation paradigm in which we estimated the target Gabor percept after adapting to sinusoidal-grating adapters. Prolonged viewing of one grating (the adapter) leads to a perceptual tilt in a subsequently presented grating (the target) in a direction opposite of the adapting orientation (the orientation shift). This tilt aftereffect (or TAE) was first termed by Gibson and Radner (1937) and widely discussed in the literature (see Clifford, 2014 for a recent review). To be perceived as vertical, the target must be oriented more in the direction of the adapter, which indicates a perceptual shift in the opposite direction.

In the first experiment of the present study, we compared the estimated TAE (orientation shift) observed in the target under different adapter conditions. We used three different oriented grating conditions: the pedestal adapter (center-only), the annulus adapter (surround-only), and the disk adapter (center and surround) presented in the upper right visual quadrant. By comparing the estimated orientation shift between the pedestal and the disk conditions, we can infer whether the lateral modulation from the surround is inhibitory or excitatory. In an attempt to link the magnitude of the TAE to filling-in, we asked observers to report their experience of filling-in during the surround-only adaptation (the annulus condition). A positive correlation between the magnitude of the TAE and filling-in would be supportive of a common underlying mechanism. In the second experiment, we focused only on the effects of the annulus adapter to understand more about the nature of the lateral modulation effect during perceptual filling-in. We separated the trials into those with reports of the filling-in percept and those without and estimated the orientation shift respectively. We can thus compare the TAE on trials where filling-in is perceived during adaptation to trials where it was not. We then constructed a model that incorporated divisive inhibition models for adaptation (Foley & Chen, 1997) and lateral interaction (Chen & Tyler, 2001) to account for the data.

## Methods

### Participants

Nine observers (six women) participated in the study, aged between 20 and 38 years, including one of the authors (Y.S.L.) and 8 naïve participants (P1 to P8). All observers participated in experiment 1, whereas three of them (Y.S.L., P1 and P3) also took part in experiment 2 and the fixation stability test. All have normal or corrected-to-normal vision. Informed consent was received from each individual before participation. The study protocols were approved by the University of Regensburg ethics committee (application number: 19-1591-101) and all experiments were performed according to the Declaration of Helsinki on human experimentation. Participants received monetary compensation or class credits as rewards. Observers first performed a short practice session before the formal experiment to become acquainted with the stimuli and the task.

### Apparatus

Four participants viewed stimuli on a 24-inch Sony Trinitron FW900 CRT monitor with 1024 × 768 resolution, the rest on a Dell S2417DG 24-inch LED monitor with 2560 × 1440 resolution. Both monitors had 120 Hz refresh rate and were calibrated and gamma-corrected using a spot photometer (MINOLTA CS-100). Mean luminance was 37.8 cd/m^2^ for the CRT monitor, and 73.8 cd/m^2^ for the LED monitor. Viewing distance and stimuli size were kept constant across the two viewing setups with the help of a chinrest. No significant difference in orientation shift estimation was found when we tested one observer on the two monitors; thus, we collapsed data across both settings for the analysis. The main experiment was carried out in a dimly lit room.

To determine if there was a difference in fixation stability across different adapter conditions, a fixation stability test was conducted in another dimly lit room with the video Eyetrace 3.20 eye tracker controlled by the video Eyetrace toolbox 3.250 (Cambridge Research Systems Ltd.). The stimuli were presented on a 60 Hz Dell 1908FP monitor with a resolution of 1024 × 768 and a viewing distance of 60 cm. The mean luminance was 30.3 cd/m^2^.

### Stimuli

**Figure. 1.**
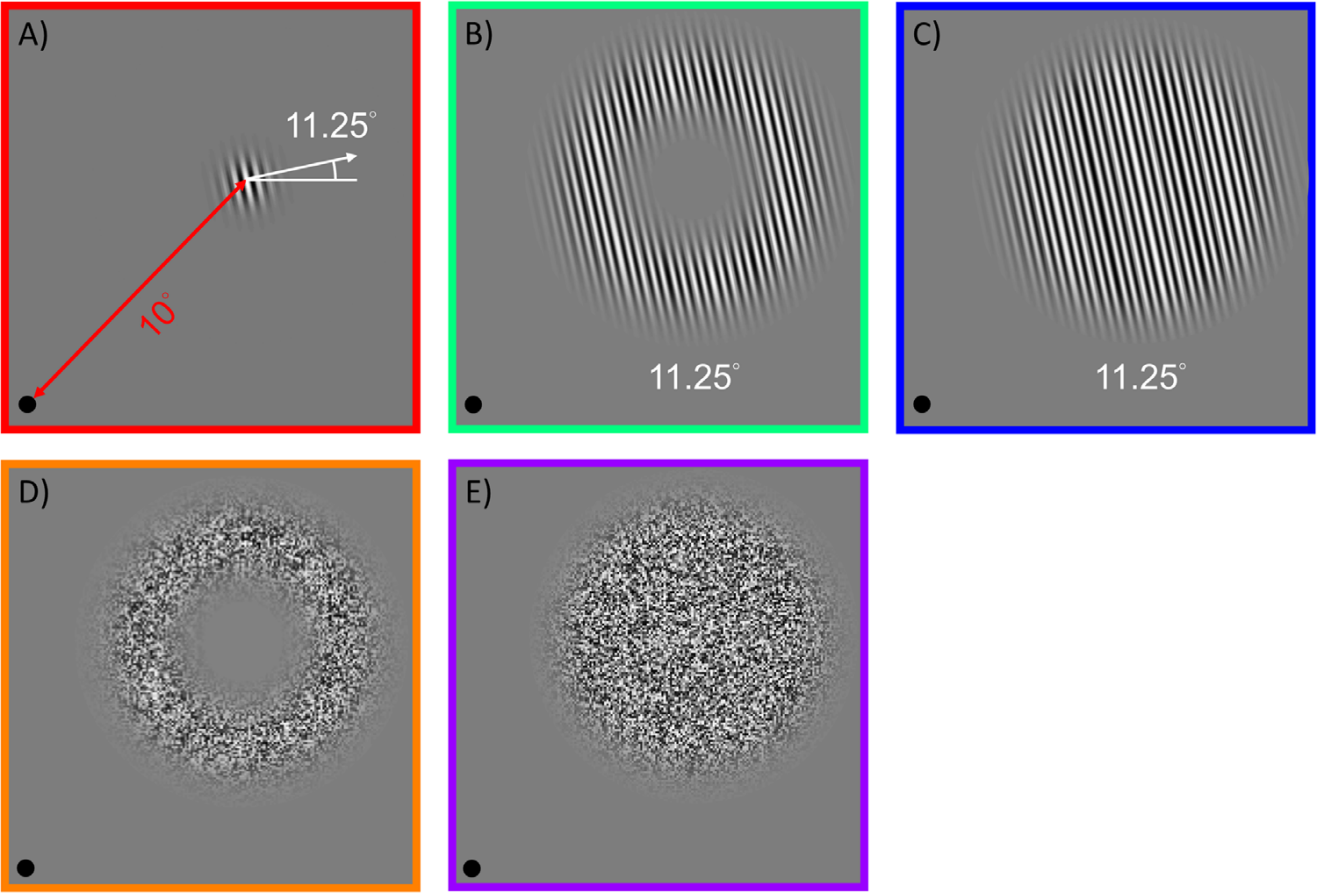
Examples of the adapters used in the current study. (**A**) Demonstrates the 11.25 degrees pedestal adapter, which was placed 10 degrees away from the fixation point, indicated by the black dot on the lower left corner. (**B**) Shows the 11.25 degrees annulus adapter, (**C**) the 11.25 degrees continuous disk adapter, (**D**) the noise annulus adapter and (**E**) the noise disk adapter. The target Gabor was of the same size as the pedestal adapter in **A**. We blurred both edges (inner and outer) of the annulus in **B** by multiplying the sinusoidal grating with a Gaussian function centering at 3.5 degrees eccentricity from the adapter center (10 degrees from the centrally located fixation point, depicted in the lower left corner in panels **A** through **E**. The Gaussian function had a scaling factor (σ) of 0.9 degrees along the inward and outward radial directions. To avoid physical overlap between the annulus and the target, we added a mean luminance disk with the radius that was 10’ larger than the Gabor target. The border color matches that used in Figure 2 (psychophysics data and model fitting results) for sake of illustration only and was not present in the experiments.

Three sinusoidal grating adapters were used in the current study (see Figure 1). A) one with the same spatial extent of the target, labeled pedestal adapter, B) an annulus adapter with no spatial overlap with the target, and C) a continuous disk adapter the same size as the annulus adapter (with no central gap). Both the pedestal adapter and the target Gabor were defined by the following equations:

$$G\left(x,y\right)=B+BCcos\;\left(2\pi fx^{'}\right)e^{\left(\frac{-x^{{}^{'}2}-{\left(y^{'}-u_{y}\right)}^{2}}{2\sigma^{2}}\right)},$$

as well as

$$x^{'}=xcos\;\theta \;+ysin\;\theta ,$$

$$y^{'}=-xsin\;\theta \;+ycos\;\theta ,$$

where *B* was the mean luminance, while *C* the pattern contrast, *f* the spatial frequency, *θ* the pattern orientation, *µ_y_* the vertical displacement of the pattern, and *σ* the scale parameter. The pedestal adapter and Gabor target had a scale parameter (*σ*) of 0.3 degrees.

The annulus adapter was created by generating a sinusoidal grating multiplied by a Gabor envelope in polar coordinates, centered at 3.5 degrees eccentricity with a scale parameter (*σ*) of 0.9 degrees. In the center of the annulus, was a disk of a mean luminance resembling an artificial scotoma. The radius of the disk was 10’ larger than the target Gabor to avoid any spatial overlap between the target and the annulus. The spatial extent of the Gabor was defined as the point when the envelope amplitude decreased below 0.5% of maximum amplitude. The disk adapter had the same outer radius as the annulus. All adapting patterns were at -1 dB (89.13%) luminance contrast and had 3 cycles per degree (cpd) spatial frequency. The stimulus orientation was defined by the direction of the luminance contrast variation; thus, the vertical grating was assigned the value of 0 degrees. The sinusoidal grating adapters were presented in one of five orientations (0 degrees, 11.25 degrees, 22.5 degrees, 45 degrees, and 90 degrees), three of which were tilted counterclockwise (CCW) from vertical and one is horizontal.

In addition to adapters with orientation information, we added three control adapter conditions without orientation information: a noise annulus (D) in Figure 1), a noise disk (E) in Figure 1), and a gray control condition in which a blank field (no adapter) was presented during the adaptation phase. We created the noise annulus and disk by randomly assigning luminance value to every 2-by-2 pixel square, resulting in a salt-and-pepper type of noise pattern (see Figure 1E). The luminance of each square was determined by *B* × (1 + *C* × *U*(*x*,  *y*)), in which *B* is the mean luminance, *C* the contrast parameter set to 89.13%, or -1dB, and *U*(*x*, *y*) a uniform distribution with a range of -1 to 1. The gray control blank-field/no adapter condition was included (not shown in Figure 1) to estimate any baseline orientation shift without prior adaptation.

All stimuli were presented 10 degrees eccentric from the central fixation on the upper right quadrant of the visual display (7.07 degrees from fixation in *x* and *y* direction). The visual stimuli were all generated using Matlab (Mathworks, Inc., Natick, MA, USA) with PsychToolbox (http://psychtoolbox.org/).

## Procedure

### Experiment 1

We used a single interval binary-choice task to estimate the target orientation shifts after adaptation to the adapting stimuli. On each trial, one of the three above-mentioned adapters were displayed for 8 seconds, with a 5 Hz counterphase flickering frequency to minimize after-image formation. The target was presented for 200 ms after the adapter, with an 83.3 ms inter-stimulus interval (ISI) between the two stimuli. Participants were asked to judge whether the target was tilted in the CCW or clockwise (CW) direction relative to vertical by pressing corresponding keyboard buttons. An auditory feedback was given to the participant after his/her response in each trial. The next trial began after the participant gave a response. We used the Ψ threshold-and-slope-seeking staircase (Kontsevich & Tyler, 1999) to determine the orientation of the next target and estimated the orientation when the observer judged the target orientation as appearing to be in the same direction as its physical appearance at an 86% rate for both CCW and CW trials. We measured the CCW and CW orientation shifts using two independent interleaved staircases with random trial sequences. When the participant judged the target orientation as the same as its physical orientation in a previous trial of the same sequence, the target orientation in the next trial in the same sequence decreased (i.e. toward the vertical orientation). Otherwise, the target orientation increased.

All 18 conditions (3 adapter types × 5 orientations plus 3 control conditions – no noise, annulus noise, full disk noise) were repeated at least three times, resulting in a total of 54 blocks, each containing 72 trials including 2 practice trials at the beginning of each block. In a random order, half of the trials contained CCW-oriented targets, the other half CW-oriented ones. A 7-point Likert-type query appeared after every 10 trials in every condition requesting the observer to retrospectively report their filling-in experience during adaptation. During the annulus adapting condition, participants pressed 1 when they experienced no perceptual filling-in on the last trial, and 7 when the central aperture was perceived as completely filled-in by the surround pattern during adaptation. They were asked to always press 1 in the pedestal and control conditions, 7 in disk conditions, when the query to do so appeared.

To make sure participants exhibited steady fixation, we included a central color detection task, in which they were asked to press the space bar whenever the fixation cross turned red during the adaptation period. All participants were trained to obtain a high fixation task accuracy (over 95% performance on the fixation task) before real data collection began.

### Experiment 2

Provided the possibility that not all trials in the annulus condition of experiment 1 induced filling-in, the orientation shift estimated might be a mixed effect from filling-in and non-filling-in trials. To further examine the effect of perceptual filling-in, we designed a complementary filling-in experiment. In experiment 2, only the annulus adapters with five orientations were used. The task in experiment 2 was identical to that of experiment 1 except that a Yes/No filling-in query was prompted after every trial, and that the program stopped only when all four conditions (CCW/CW orientations × with filling-in/without filling-in) reached at least 35 trials each (not including the practice trials). We estimated the orientation shift necessary to null the TAE in each of the four conditions based on the target orientation presented and the observer binary-choice response in each trial using the same threshold-and-slope estimation algorithm as the Ψ procedure implemented in our experiment (Kontsevich & Tyler, 1999). We took the data in the first 35 trials of each condition so that the amount of trials used to estimate the perceived orientation shift was kept the same across all conditions. By doing so, we can separately examine the adaptation effect on the target with and without reports of perceptual filling-in.

### Fixation stability test

To make sure that the difference in TAE between different adapter conditions did not result from differences in how well the participants fixated, we conducted a control experiment with an eye tracker to estimate the potential effects of fixation stability on the results. We recruited three observers who participated in both experiments 1 and 2 (including one author). Each participant performed one run of the following four conditions: the pedestal adapter, the annulus adapter, and the disk adapter in experiment 1, and the annulus adapter in experiment 2. Participants completed the four conditions in random sequence. The experimental procedure was exactly the same as that in experiments 1 and 2, respectively, except that an eye tracking calibration was performed at the beginning of each run.

## Results

**Figure 2.**
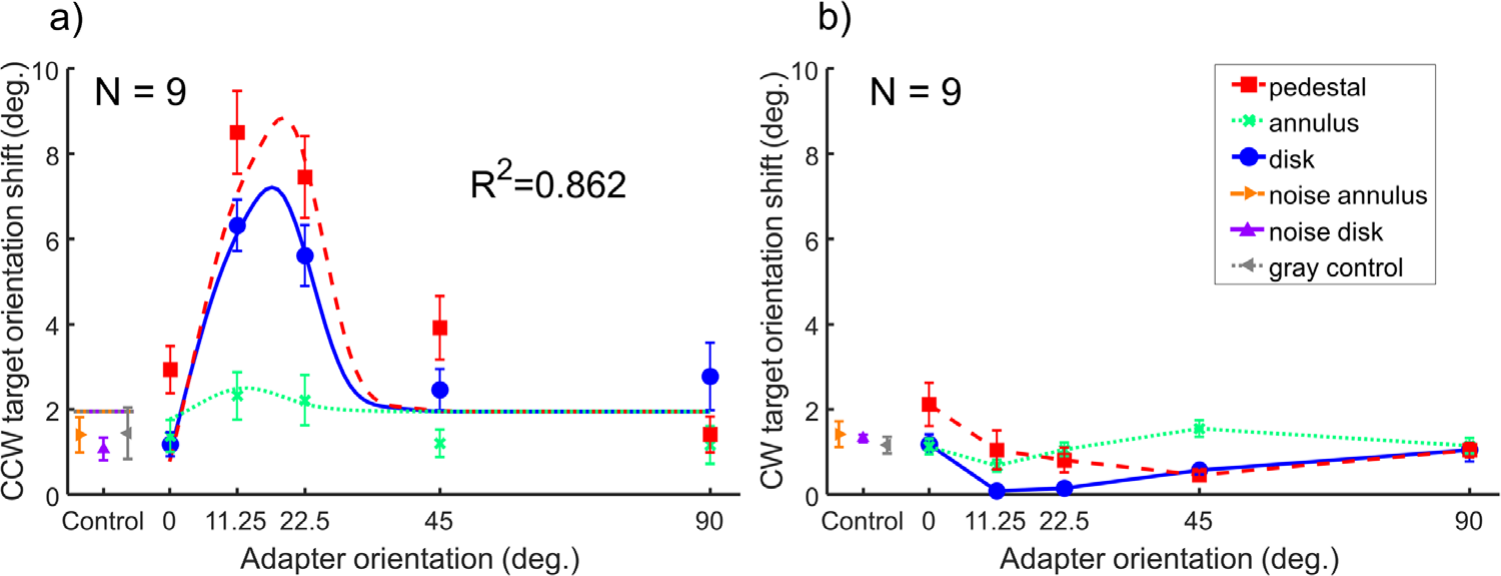
The averaged data of estimated perceived orientation shifts in experiment 1. Two types of targets were presented after adaptation: a CCW-oriented target (**a**) and a CW-oriented target (**b**). We estimated how much orientation shift of each type of target was needed to cancel the TAE induced by the adapter. In the current study, all adapters were of CCW orientation relative to the vertical orientation. In both CCW **a** and CW target **b**, the three curves show results of oriented grating adapters (red-dashed curve: pedestal adapter; green-dotted curve: annulus adapter; blue-solid curve: disk adapter), with five orientation levels indicated on the x-axis. The three triangle markers present data points from the three control conditions (orange right-pointing triangle: noise annulus adapter; purple up-pointing triangle: noise disk adapter; gray left-pointing triangle: gray control/no adapter condition). The error bars are ±1 standard error of measurement. In **a**, the smooth curves are the best fits of our computational model. The short-dashed lines close to the three control data represent model fits of the control conditions (see Model part in Results).

In the following sections, we will focus our discussion on the averaged data across all observers in experiments 1 and 2. The estimated values of Ψ procedure of each participant in each run in the two experiments are shown in Supplementary File S1 (with observer LYS referred to as P0) in .xlsx (Excel; Microsoft, Redmond, WA) format. The orientation shift of both CCW and CW target is expressed in degree away from vertical, whereas the unit of the slope is *d*′/log θ, detectability per log target orientation (in degree). In experiment 1, we estimated the target orientation shift necessary to null the TAE under different adapter conditions. We had three oriented grating adapters: the pedestal adapter (center-only), the annulus adapter (surround-only), plus the disk adapter (center and surround). In addition, we included three control adapters that contain no orientation information: the noise annulus adapter, the noise disk adapter, and the gray control condition (no adapter during the adaptation period). The averaged data of nine observers in experiment 1 are shown in Figure 2 (for individual-subject data, see Supplementary Figure S1). Two types of target were presented in random sequence after the adapter: a CCW rotated target or a CW rotated target. We estimated how much orientation shift away from vertical of each type of target was needed to cancel the TAE induced by the adapter. The perceived orientation shifts of the CCW target are plotted against adapter orientation in Figure 2a, whereas that of the CW target are shown in Figure 2b. The CCW orientation shift varied with adapter type and adapter orientation, whereas the CW orientation shift remained mostly unchanged. This was expected because most of our adapters were oriented in the CCW direction and created a CW TAE that led to a robust perceived CCW orientation shift that was captured best by the CCW targets. The CW-oriented targets were included in the experiment as catch trials to make sure the observers were following task instructions. Thus, the data demonstrated in Figure 2b can be seen as the orientation shift in a control condition when no significant TAE was induced. In the following sections, we focus only on the CCW orientation shift results of the CCW target.

For the oriented grating adapters, the amplitude of perceived orientation shift (compared to the gray control baseline) was highest in the pedestal condition (red-dashed curve), intermediate in the disk condition (blue-solid curve), and smallest in the annulus condition (green-dotted curve). We performed paired *t*-tests between the peaks (at 11.25 degrees) of oriented grating adapter conditions and the gray control condition and found that all three perceived orientation shifts were significantly higher than the baseline. The *t*(8) was 7.53 for the pedestal, 7.44 for the disk, and 3.07 for annulus conditions with a Bonferroni-corrected *P* value of < 0.001 for pedestal and disk (effect size Cohen's *d* = 2.91 and 2.69, respectively) and .024 for the annulus (Cohen's *d* = 0.50) conditions.

For the noise annulus and noise disk adapters, no orientation shift variation was found compared with baseline. The *t*-tests between the noise stimuli and the gray control were not significant (*t*(8) = -0.91 for noise disk, -0.16 for noise annulus, n.s.). This suggests that when the adapter carried no coherent orientation information, the target orientation percept remained unchanged after adaptation. These results suggest that the observed adaptation effect was orientation specific. In the following, we will focus mainly on the effects of the oriented grating adapters.

In Kontsevich and Tyler (1999), the authors performed Monte-Carlo simulations to examine how the threshold and slope estimated by the Ψ method would converge after a certain amount of trials. Figure 1 of their paper shows that the estimated threshold reached a precision of 2 dB after about 30 trials, whereas the estimated slope required about 300 trials to reach the same precision level. In the current study, only 35 trials were presented in each adapter condition, which was sufficient for threshold convergence but not for slope convergence. As a result, the estimated slope values stayed around the initial value two, which was the middle point of the slope range, set from 1 to 4 in our study (for the Ψ method slope values, see Supplementary File S1). Thus, we will not discuss the Ψ slope values below.

The Ψ method was chosen as a placement method in the current study, allowing us to use an adaptive staircase method to determine which test orientation to be used based on the observer previous response. However, the Ψ staircase method was initially developed to estimate the threshold and slope in a 2AFC task, not in a binary-choice task used here. In addition, the Ψ method assumed that at the lowest stimulus level, the correct probability corresponds to 0.5. Such assumption might not hold in our case (especially after adaptation), resulting in deviation of estimated parameters from the actual response probability. To examine whether the estimated orientation shift reflects the 86% CCW responding rate, we fitted a cumulative normal psychometric function (PF) to the raw data (the CCW trials) for each participant with the Matlab-based Palamedes toolbox (Prins & Kingdom, 2018). We then examined the relationship between the estimates of the PF fitting results and the ones from the PSI method. The estimated orientation shift required to reach the 86% CCW responding of the two methods were found to be quite comparable within each participant. To examine whether there was a systematic relationship between the orientation shift and the slope (a possible confound between the bias and unreliability in the data), we calculated the Pearson correlation coefficient between the estimated 86% CCW responding rate and the fitted slope (beta) value in the PF fitting results of all conditions for each participant. Results showed that the orientation shift was negatively correlated with the slope in two out of the nine participants (*rs*(14) = -0.51 and *rs*(14) = -0.50, *P* = 0.021 and 0.024), suggesting that in the data of these two observers, the bias and unreliability were proportional to each other, but not in the data of the remaining seven observers. Details of the PF fitting methods, the comparison between two methods, as well as the correlation between the PF fitting parameters can be found in Supplementary File S2.

Another way of examining the data in the current study is by combining both CW and CCW trials of the same adapter condition together and fitting one PF function to the combined data set. The procedure and results of such data reanalysis is included in the Supplementary File S2, whereas the raw trial data used to fit the PF functions are shown in Supplementary File S3. The results show a very similar pattern as presented in Figure 2a (the estimated orientation shift by the Ψ method). Thus, in the remaining sections of this paper, we will focus our discussion on the Ψ method threshold estimate (the orientation shift).

Lateral modulation can express itself either as lateral excitation or as lateral inhibition. If the adapter surround induced lateral excitation effect on the adapter center, then after adaptation we should expect an increased adaptation effect. On the other hand, if the adapter surround imposed lateral inhibition instead, we should observe a decrease of the adaptation effect on the target. Our results showed that the CCW orientation shift in the full disk condition was lower than for the pedestal condition, suggesting that lateral inhibition arose when the surround was stimulated during adaptation.

**Figure 3.**
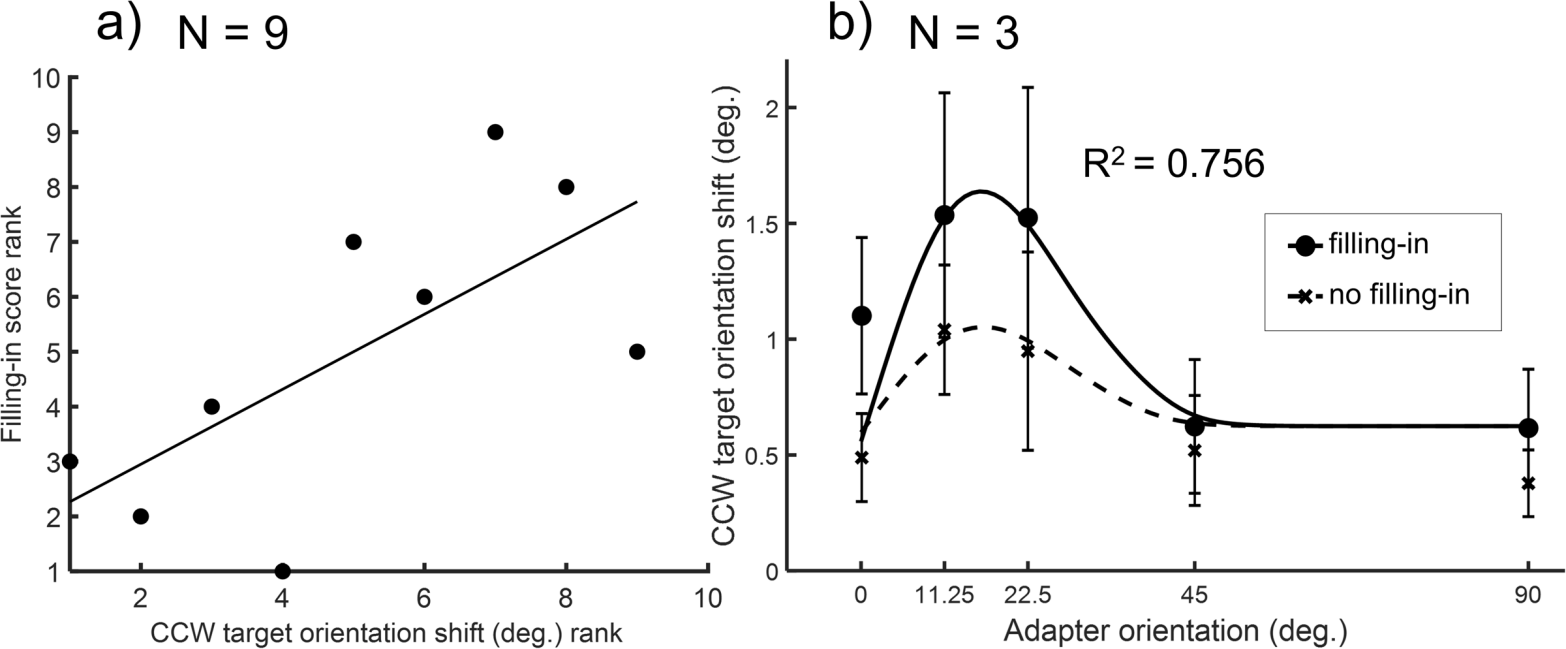
The relationship between the perceptual filling-in (occurring in the annulus adapter condition) and the orientation shift (TAE). (**a**) The scatter plot of the correlation between the rank of the CCW target orientation shift and the rank of the filling-in score of the annulus condition of each participant (*N* = 9) in experiment 1. The solid line represents the least-squares fitted regression line, *rs*(7) = 0.69. (**b**) The averaged data of estimated perceived orientation shifts of CCW target in experiment 2 (in which only the annulus adapter was used). The symbols (disk: with filling-in; cross: without filling-in) represent the behavioral data while the smooth curves (solid curve: with filling-in; dotted curve: without filling-in) the best fits of the model (see Results). The error bars are ±1 standard error of measurement.

To evaluate the association between the filling-in percept during adaptation and the perceived orientation shift during test, we calculated the non-parametric Spearman's rank correlation coefficient between the filling-in reports (see Methods for details) and the perceived orientation shift of the annulus condition at its peak (11.25 degrees). The result (shown in Figure 3a) showed a positive correlation coefficient that was significant (*rs*(7) = 0.69 *P* = 0.022), indicating that across all observers the stronger the perceptual filling-in perceived by the observer, the stronger the adaptation effect induced for the target.

In experiment 1, we did not monitor the subjective perceptual filling-in experienced by the observers on a trial-by-trial basis. Instead, the query was only prompted once every 10 trials. As a result, we could only access the averaged filling-in percept in the whole block and the estimated orientation shift might not entirely reflect the effect when filling-in occurred. Given that the central area could undergo a different amount of adaptation effect with or without filling-in, in experiment 2, we asked the participants to report whether they experienced perceptual filling-in during adaptation in the annulus condition immediately after each trial. This way, we could separately estimate the adaptation effect for filling-in and non-filling-in trials.

The data of three observers in experiment 2 are presented in Figure 3b (individual-subject data in Supplementary Figure S2). Compared with the data when filling-in was not reported, the orientation shift was larger when filling-in occurred. A 2-way repeated measure ANOVA was conducted on the averaged data across the three observers to compare the main effects of filling-in and five adapter orientations, as well as the interaction between these two factors. The filling-in main effect was significant (*F*(1, 18) = 15.11, *P* < 0.01, $\hat{f}$ = 0.69), suggesting that the TAE was stronger when filling-in was perceived during adaptation. The main effect of adapter orientation was also significant (*F*(4, 18) = 9.99, *P* < 0.01, $\hat{f}$ = 1.09), suggesting that the TAE varied across different adapter orientations. The interaction between filling-in and adapter orientation was not significant (*F*(4, 18) = 0.92, *P* = 0.48) suggesting that the orientation-tuning of the aftereffect was not altered by filling-in. The results agree well with the positive correlation across different subjects between the filling-in score and target orientation shift observed in experiment 1 (see Figure 3a).

To verify whether the observer's fixation differed across different adapter condition, we estimated the fixation stability of the three participants with an eye tracker. The video device records the horizontal and vertical eye positions during the stimuli presentation on each trial in the following four conditions: the pedestal adapter, the annulus adapter, and the disk adapter in experiment 1, and the annulus adapter in experiment 2 (in which results are sorted into filling-in and no filling-in trials based on the subjectively reported filling-in percept during adaptation). All adapters used in the fixation stability test had the same (11.25 degrees) orientation. We preprocessed the raw eye tracking data by removing timepoints with missing data (e.g. when observer blinked during the stimulus presentation) and ruling out eye positions that surpass 3.3 degrees in amplitude (10 times the normal range of microsaccade, which is usually less than 20’ [Carpenter, 1988]) in radius to exclude outliers and potential recording artifacts.

**Figure 4.**
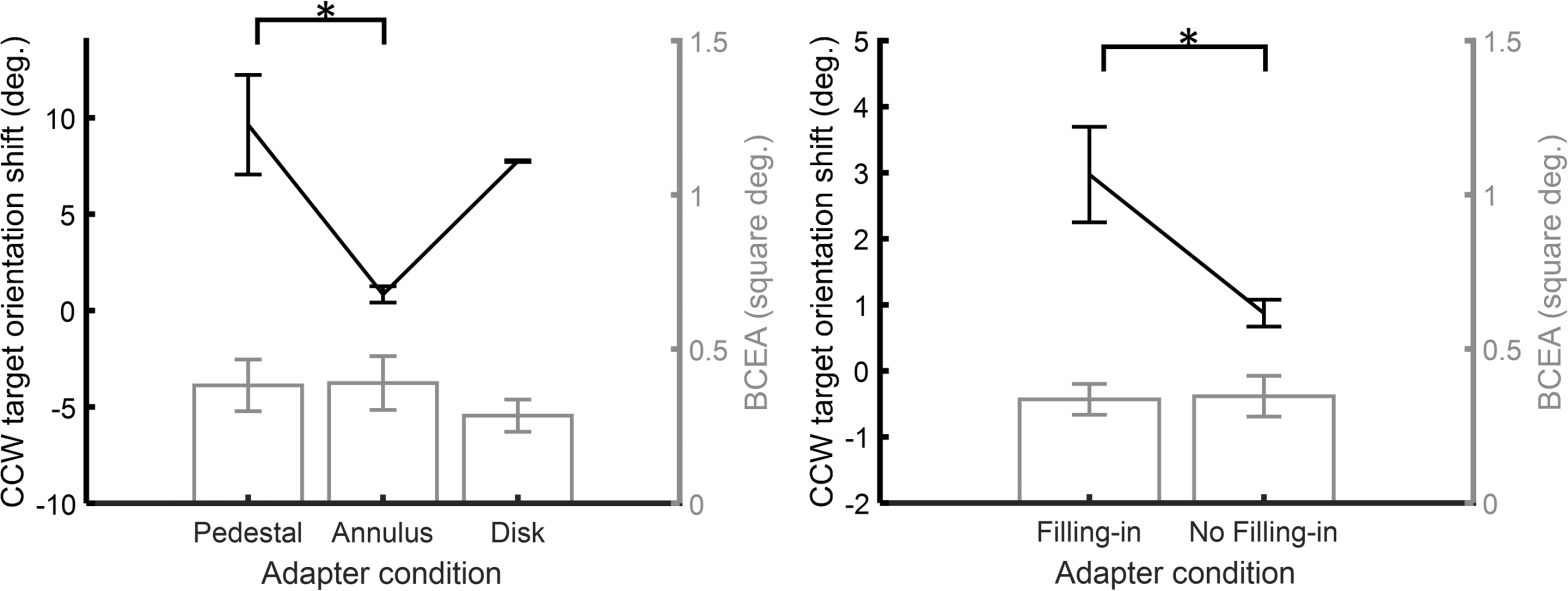
The averaged CCW target orientation shifts (TAE) and BCEA values across three participants in different adapter conditions. In both panels, the line represents the orientation shift plotted against the adapter condition with value and unit corresponding to the left y-axis, while the bars represent the BCEA plotted against the adapter condition with reference to the right y-axis. The left panel shows the three conditions in experiment 1: the pedestal, the annulus, and the disk adapters. The right panel shows the two conditions in experiment 2: the filling-in and no filling-in conditions (both with the annulus adapter). The error bars depict ±1 standard error of measurement. The asterisk symbol demonstrates which two conditions/trial-types are significantly different (*P* < 0.05).

We estimated the fixation stability of each trial by calculating the bivariate normal ellipse area (BCEA) value in each trial defined by the following equation (Castet & Crossland, 2012; Schönbach, Ibrahim, Strauss, Birch, Cideciyan, Hahn, & Sunness, 2017),

$$BCEA=2k\pi \sigma_{H}\sigma_{V}{\left(1-\rho^{2}\right)}^{0.5},$$

where σ_*H*_ and σ_*V*_ are the standard deviations of the horizontal and the vertical fixation positions, and ρ the Pearson's correlation coefficient between the two fixation positions. *k* is a constant determining the probability area as in

$$P=1-e^{-k},$$

in which *e* is the base of the natural logarithm and *P* the probability area. We used a *k* value of 3.079, which leads to a *P* of 0.954. Figure 4^5^ demonstrates the averaged orientation shift of the CCW target and the BCEA value across the three participants in different adapter conditions.

**Figure 5.**
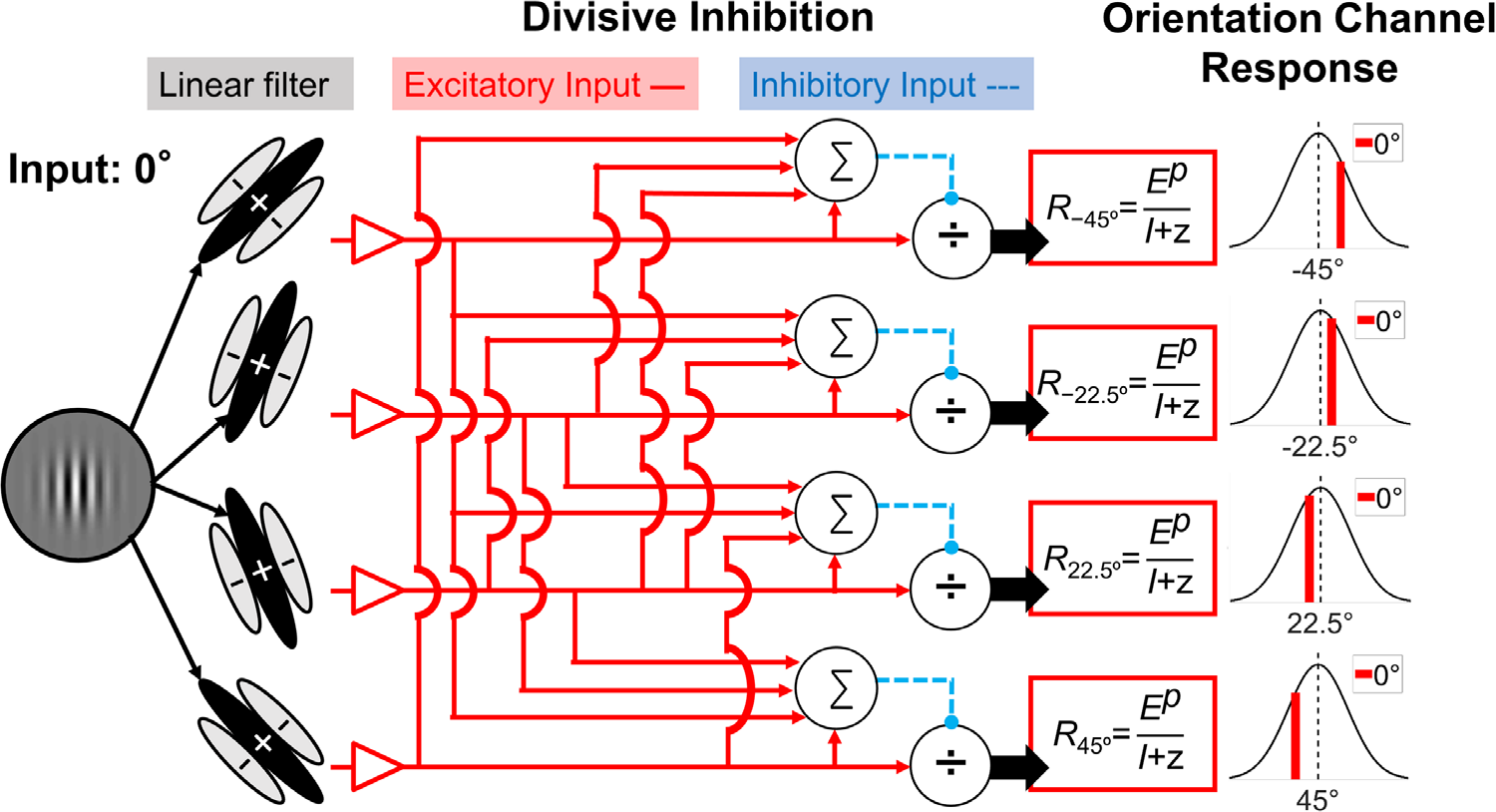
The schematic diagram of the model used in the current study. The orientation channel response to the target is determined by an excitatory component (*E*) raised by a power (*p*) then divided by an inhibitory component (*I*) plus a constant (*z*). See text for detailed description.

One-way repeated measure ANOVAs on the orientation shifts and the BCEAs in experiment 1 data were conducted. The 1-way ANOVA conducted on the mean orientation shift shows that there is a difference between different adapter types (*F*(2, 4) = 10.93, *P* < 0.05, $\hat{f}$ = 0.62). The pairwise post hoc Tukey HSD (honestly significant difference) multiple comparison test indicates that the pairwise contrast between the pedestal and the annulus (*qT*(3,6) = 6.28, *P* < 0.05) exceeds the critical difference, whereas the contrast between the pedestal and the disk (*qT*(3,6) = 1.34, *P* = 0.64) as well as the contrast between the disk and the annulus (*qT*(3,6) = 4.93, *P* = 0.05) did not. In contrast, the 1-way ANOVA computed for the BCEA values revealed no significant difference between the adapter types (*F*(2, 4) = 4.40, *P* = 0.10). Again, the paired *t*-test done on the perceived target orientation shifts of the two conditions in experiment 2 shows that there is a difference between the two (*t*(2) = 3.95, *P* < 0.05), whereas the *t*-test on the BCEA values when filling-in was compared with when it was not reported failed to show a significant difference (*t*(2) = -2.16, *P* = 0.16). These statistics suggest that there was no significant difference between the fixation stability across different adapter types, although they induced different magnitudes of the TAE. Nor did we find evidence for differences in fixation stability on trials where subjects perceived filling-in compared to when they did not.

## Model

### Model architecture

Contrast gain control or divisive inhibition has been proposed to explain a wide range of visual phenomena and can be observed as early as the processing stage of the retina. This normalization process has also been identified in areas such as in LGN (Bonin, Mante, & Carandini, 2005) and primary visual cortex (Albrecht & Geisler, 1991; Gardner, Sun, Waggoner, Ueno, Tanaka, & Cheng, 2005; Heeger, 1992; Müller, Metha, Krauskopf, & Lennie, 2003; Ohzawa, Sclar, & Freeman, 1985). Wilson and Humanski (1993) used a contrast gain control model to explain the contrast threshold elevation after adaptation to cosine gratings of selected spatial frequencies. Their model could account for the TAE data from Campbell and Maffei (1971). Similarly, Foley and Chen (1997) proposed a two-process divisive inhibition model, in which the adaptation affected up to two parameters in the denominator, to interpret the pattern adaptation effect on target contrast threshold. Meese and Holmes (2002) proposed an alternative gain control model (where one adaptation factor was introduced in the denominator) to explain their own masking data and the adaptation data of Foley and Chen (1997).

We used a variant of divisive inhibition (or contrast normalization) model to fit our TAE data. Our model is inspired from both Foley and Chen (1997), in which the adaptation effect was captured by increases in the parameters in the denominator, and Chen and Tyler (2001), in which the lateral modulation effect from the flankers was represented by multiplicative parameters. Figure 4 demonstrates a schematic diagram of the current model. In the current model, we assumed multiple orientation channels (as local mechanisms in one hyper-column) with preferred orientations distributed evenly from -90 degrees to 90 degrees (with CW orientation assigned negative values, and CCW positive values) degrees at intervals of 11.25 degrees. The tuning bandwidth (full width at half maximum, or FWHM) of all channels was set at 22.5 degrees. The response of each oriented channel toward the target first goes through a receptive field-like linear operator (the linear filter in Figure 4) then a nonlinear operator. The final response predicted by the model is determined by the excitatory component divided by the inhibitory component and an additive constant.

The excitation of the linear operator in channel *j* toward the target is determined by the product of the sensitivity profile of the linear operator and the input image (Chen, Foley, & Brainard, 2000; Foley & Chen, 1999; Phillips & Wilson, 1984). Here, we assume the profile to be a Gabor function and the input image to be also a Gabor pattern (see section Stimuli in Method). The input image could be either the target or the adapter. The product of the Gabor function and the Gabor pattern, integrated over space, can be factored into the following components: (1) because the input contrast, *C_i_* of the *i^th^* image component (such as the target, or one of the adapters), is independent of the spatial structure, it can be taken out as an individual term. (2) The orientation dependent component that can be captured by an orientation tuning function, *O_j_*, and (3) the orientation independent part of product that can be taken as a constant in our experiment. We called this constant the sensitivity parameter, *Se_i_*. The excitation of the *j*^th^ channel to the *i*^th^ image component can thus be defined as,

(1) $$E_{ij}^{'}=Se_{i}\cdot C_{i}\cdot O_{j}\left(\theta_{i}\right).$$

The orientation tuning function, in our case, is assumed to be a Gaussian function, as has been implemented in studies modeling neuron tuning curves and psychophysics data (Deneve, Latham, & Pouget, 1999; Paradiso, 1988; Pouget, Dayan, & Zemel, 2000; Pouget, Zhang, Deneve, & Latham, 1998; Schwartz, Hsu, & Dayan, 2007; Westrick, Heeger, & Landy, 2016; Wilson & Humanski, 1993). That is,

(2) $$E_{ij}^{'}=Se_{i}\cdot C_{i}\cdot e^{-\;\frac{{\left(\theta_{i}-\theta_{j}\right)}^{2}}{\sigma^{2}}},\;$$

where the θ_*j*_ is the channel preferred orientation and σ^2^ the channel variance, which determines the channel bandwidth.

The linear operator excitation in Equation 2 is then halfwave-rectified (Chen & Tyler, 2001; Chen & Tyler, 2002; Foley, 1994; Foley & Chen, 1997; Foley & Chen, 1999) into

(3) $$E_{ij}=max\left(E_{ij}^{'},\;0\right),$$

where max represents the operation to select the larger to the two numbers. Without adaptation, the channel response to the *i*^th^ input image is computed by the rectified excitation raised by the power *p* then divided by the inhibitory component *I_ij_* as well as the normalization constant *z*. That is,

(4) $$R_{ij}=\;\frac{{E_{ij}}^{p}}{I_{ij}+z}\;.$$

The inhibitory component is the summation of all relevant mechanisms (in our case, *N* channels), given by

(5) $$I_{ij}=Si_{1}\cdot {\left(E_{ij}\right)}^{q},$$

where *Si*_1_ is the inhibition sensitivity of the *j*^th^ channel (self-inhibition).

The perceived orientation is determined by a population coding operation, which is the preferred orientation of each channel, θ_*j*_, weighed by the response of that channel, *R_ij_*, divided by the sum of responses of all channels (Clifford, Wyatt, Arnold, Smith, & Wenderoth, 2001; Deneve, Latham, & Pouget, 1999; Jin, Dragoi, Sur, & Seung, 2005; Mély, Linsley, & Serre, 2018; Pouget, Zhang, Deneve, & Latham, 1998; Westrick, Heeger, & Landy, 2016) and the adjusted by an internal bias parameter, *m*. That is

(6) $$P_{i}=\;\frac{\sum_{j=1}^{N}R_{ij}\cdot \;\theta_{j}}{\sum_{j=1}^{N}R_{ij}}+m.$$

The internal bias parameter is needed because the observers might make a CW or CCW match to the target even in the control conditions void of experimental manipulation.

### Modeling implementation

In our experiment, each trial started with prolonged exposure to an adapter. In adaptation, the visual system adjusts its response characteristics to accommodate the current visual environment (Barlow, 1972; Barlow & Földiák, 1989). Indeed, the dynamic range of the contrast response function of V1 neurons can be changed following a prolong exposure to a stimulus (Albrecht, Farrar, & Hamilton, 1984; Anderson, Barlow, Gregory, Carandini, Barlow, O'keefe, & Movshon, 1997; Gardner, Sun, Waggoner, Ueno, Tanaka, & Cheng, 2005; Sclar, Lennie, & DePriest, 1989). Such dynamic range shift can be modelled with a change in the semi-saturation parameter of a Naka-Rushton (Naka & Rushton, 1966) type model of contrast response function. In our model, a shift of the dynamic range can be achieved by a change in parameter *z* in Equation 4.

In psychophysics, Foley and Chen (1997) found that, in addition to the additive constant (*z* in Equation 4), the sensitivity of the target mechanism to the inhibitory signals from the mechanisms that tune to the orthogonal orientation also increased after adapting to a Gabor pattern. The cross-orientation inhibition can be captured by a term in the inhibition part, which is the summation of the excitations across channels. The experiments of Foley and Chen (1997) systematically varied the contrast of the cross-orientation components of the image and thus the cross-orientation inhibition to the response function. In our experiment, all the stimuli had the same contrast. As a result, the sum of the responses across orientation channels, and in turn the cross-orientation inhibition, would be similar for targets of any orientation and thus can be absorbed by the additive constant, *z*. Therefore, for the current experiment, we only need to consider the change of the additive constant *z* following the adaptation. That is, after adaptation, the response function in Equation 5 becomes,

(7) $$R_{ij}=\;\frac{{E_{ij}}^{p}}{I_{ij}+z_{j}^{'}}=\;\frac{{E_{ij}}^{p}}{I_{ij}+\;z\cdot a_{j}},$$

and

$$a_{j}=\left(1+R_{kj}\right),$$

in which *R_kj_* is the response of channel *j* to the *k*^th^ adapting stimulus, which can be computed in the same way as the response with Equations 1 to 5. Notice that there were different types of adapters in our experiment. Thus, we allowed the excitatory sensitivity to each type of adapter, *Se_i_*, to be a free parameter.

We fitted the model to the group averaged data (as shown in Figures 2 and 3) with a Powell's algorithm (Press, Teukolsky, Vetterling, & Flannery, 1988) to search for the parameter values that minimize the sum of the squared differences between the measured and predicted TAE reported for the target, or sum of squared error (SSE). The set of the best fit parameters is shown in Table 1. The smooth curves in Figures 2 and 3 represent the fits.

**Table 1.** List of fitting parameters and R^2^ for the averaged CCW orientation shifts of orientation-grating stimuli and the gray control/no adapter condition.

Parameter		
*Se*		
	Target	**100.00**
	Pedestal	**100.00**
	Annulus	1.01
	Disk	38.7900
	Control^*^	**0.00**
	Filling-in	**10.00**
	No filling-in	1.22
*Si*_1_	Exp 1	0.38
	Exp 2	0.74
*p*	Exp 1	1.26
	Exp 2	0.77
*q*	Exp 1	0.35
	Exp 2	0.41
*z*		**0.50**
σ		**9.56**
*m*	Exp 1	-1.92
	Exp 2	-0.63
*R^2^*	Exp 1	0.862
	Exp 2	0.756
RMSE	Exp 1	0.90
	Exp 2	0.20

*Notes*. Fixed parameters are marked with bold font. The same set of parameters were used to fit the three control conditions (the noise annulus, the noise disk, and the gray control) in experiment 1. Six free parameters were used to fit the data of experiment 1 (*Se* in the annulus and the disk conditions, *Si*_1_, *p*, *q*, and *m*) and experiment 2 (*Se* in the filling-in and no filling-in conditions, *Si*_1_, *p*, *q*, and *m*).

For experiment 1, the model can explain up to 86.2% of the variance in the averaged data. The root mean square error (RMSE) was 0.90, slightly larger than the mean standard error of measurement, which was 0.56. For experiment 2, the model can explain 75.6% of the variation in the averaged CCW data. The RMSE was 0.20, compatible with the mean standard error of measurement, which was 0.32.

Table 1 shows the best-fitting parameters for both experiment 1 and experiment 2 (the best-fitting parameters of individual-subject data can be found in Supplementary File S4, a Microsoft Excel file in which each sheet contains data of all participants of one experiment). Except for *p*, *q*, *Si*_1_, *m*, and *Se* in the annulus and the disk conditions in experiment 1 as well as the *Se* in the no filling-in trials in experiment 2, all parameters were fixed (6 free parameters in total), because the goodness of fit (*R*^2^) did not change empirically if they were free parameters or fixed. For the same reason, parameter *z* can be fixed as 0.50.

The parameter *Se* reflects the channel sensitivity to the adapter *center*, which has the same spatial profile as the target. Thus, changes in *Se* represents the amount of lateral modulation introduced from the adapter surround to the adapter center. For comparison purposes, we fixed the *Se* value in the pedestal condition at 100 as the baseline in experiment 1 to compare between the lateral modulation effect in different adapter conditions. Values above 100 represent a stronger adaptation effect, whereas those below 100 a weaker effect. The lateral modulation effect induced from the adapter surround can be either excitatory or inhibitory. If the modulation effect is excitatory, then after adaptation, such lateral excitation should enhance the adaptation effect on the adapter center. If, on the other hand, the adapter surround induces an inhibitory effect, then when the surround was introduced during adaptation this lateral inhibition should lead to a decrease in the TAE. In our case, when the surround was present in the disk condition, *Se* value decreased to below 40, suggesting a significant decrement of the adaptation effect. The model fitting result agrees well with the empirical finding that the target orientation shift was less in the disk condition than in the pedestal condition, indicating a lateral inhibition effect from the surround.

To examine whether different *Se* values in the model produces a better fit, we also conducted a model comparison between (a) the full model (the current model, in which *Se* is free parameter except for the pedestal condition, leading to six free parameters) and (b) the reduced mode (when all *Se* are fixed as 100.00, thus only four free parameters left). The coefficient of determination *R^2^* was reduced substantially from 0.861 to 0.56 in the fitted results of the reduced model. An F-test between the two models showed that the full model can explain significantly more variance than the reduced model (*F*(2, 12) = 12.09, *P* = 0.0013). Such results suggested that the amount of lateral modulation from the surround to the center varied across different adapters.

To test whether the filling-in and no filling-in trials demonstrate different characteristics in model fitting, we fitted the same aforementioned model to the experiment 2 data. Here, for the purpose of comparison across conditions, we fixed the *Se* for the filling-in condition at a value of 10 and allowed the *Se* for the non-filling-in condition to be a free parameter. The best fits (as shown in Table 1) *Se* for the non-filling-in condition was much lower than that for the filling-in condition, suggesting that the adaptation effect was weaker when perceptual filling-in was not perceived.

## Discussion

To understand how the adapter stimulus affects the central target percept, we used sinusoidal adapters either covering the center, the surround, or both stimulus regions. Our results in the experiment 1 showed that the adaptation effect on the Gabor target, reflected by the TAE that shifted the perceived target orientation in a direction opposite to that of the adapter, was greatest in center-only (pedestal), intermediate in center-surround (disk), and smallest in surround-only (annulus) condition (see Figure 2a). The difference between the pedestal and the disk condition suggests that adding a surround decreases the adaptation effect of the center, indicating an inhibitory lateral modulation from the surround. Although there was no spatial overlap between the adapter and the target in the annulus condition, a significant TAE was still induced after adaptation. This finding suggests that during adaptation, the adapter surround induced the TAE at the adapter center that was vacant of physical stimulus, thereby leading to a perceived shift in target orientation. We also found a positive correlation between the self-reported strength of perceptual filling-in during adaptation and the TAE magnitude across observers. This result can be taken as evidence for adapter surround contribution to both perceptual filling-in and the adaptation effect. To better discuss the nature of the TAE and its relationship to perceptual filling-in, we turn to the results of the second experiment. In experiment 2, we estimated the orientation shift on trials when perceptual filling-in was reported and when no filling-in was reported and found that the orientation shift was larger when perceptual filling-in was experienced in the annulus adapter. Our results indicate that the perceived filling-in during adaptation reflects the strength of the subsequent TAE. These findings suggest that exposure to the adapter surround acts to alter contrast gain in the central target region during the adaptation period and this effect is more pronounced when observers experienced filling in. To account for the underlying mechanism of the adaptation effect, we propose a computational model.

The adaptation effect was modelled in the same way as Foley and Chen (1997), who suggested that the additive constant in the denominator of the response function increased after adaptation. We thus implemented the adaptation effect in a mechanism by multiplying the additive constant, *z*, in the denominator of the response function by an adaptation factor (Equation 7), which in turn was determined by its response to the adapter. The stronger a mechanism responded to an adapter, the more adaptation effect it exhibited.

The effect of different adapters was manifested as a change of just one parameter *Se* (the excitatory sensitivity). We found that *Se* was smaller in the disk condition than in the pedestal condition (see Table 1). Notice that the disk adapter had a larger size and thus potentially greater overlap with the receptive fields of the target channels than the pedestal adapter. Such reduction of sensitivity is not consistent with the effect of spatial summation (Chen, Yeh, & Tyler, 2019). Instead, it implies that the extra area of the disk provides an inhibitory signal to the target channels and makes it less sensitive to the input stimuli.

The *Se* value for the annulus condition reflects the effect from the surround in the absence of the adapter center. In the experiment 2, where only annulus adapter was used, the change of parameter *Se* reflected the TAE induced from the surround, which corresponded in magnitude to the subjective filling-in percept of the observers. Thus, our model captured the effect of the surround region that simulated both the perceived orientation shift and the perceived filling-in. However, the current model provides no direct indication as to the cause or underlying mechanism of filling-in. One possibility is that the surround region actively modulates the center filled-in region through lateral interactions (Chen, Tyler, Liu, & Wang, 2005; Komatsu, 2006; Komatsu, Kinoshita, & Murakami, 2002). Because the empirical data reported in the literature, including neuroimaging results, show evidence supporting both lateral inhibition and lateral excitation during perceptual filling-in, it could be that filling-in percept results of an interplay between the excitatory and inhibitory lateral interaction from the surround feature to the central blank regions. Alternatively, it is also suggested that the center region is filled-in due to sensitivity loss at the boundary between the blank area and the surrounding context, that is, the isomorphic theory (Cohen & Grossberg, 1984; Gerrits & Vendrik, 1970; Kinoshita & Komatsu, 2001; Neumann, Pessoa, & Hansen, 2001). Yet, another possibility is related to the idea that the brain simply “ignores” the missing information and assumes a complete surface, or the symbolic or cognitive theory (Pessoa, Thompson, & Noë, 1998; von der Heydt, Friedman, & Zhou, 2003). The limitation of the current study is that although the adaptation paradigm is useful for revealing the effect of different adaptors, it is difficult to pinpoint the exact neural activation or neural mechanism during perceptual filling-in even with the help of a model. In our current model, the value of parameter *Se*, excitatory sensitivity to the adapter center, reflects the strength of TAE, and perceived filling-in induced by the annulus adapter. However, the fact that the variation in *Se* could be the result of many possible factors, such as differences in lateral interaction, different levels of induction from mechanism sensitive both to the center and surround, changes in boundary sensitivity, and even some top-down influences from higher brain regions, we could not determine the source of the filling-in perceived by observers in the current study. Such a limitation could be overcome by implementing neuroimaging methods, such as fMRI in future studies.

To make sure that the difference in TAE between different adapter conditions did not originate from differences in how well the participants maintained their fixation, we conducted a control experiment to estimate the fixation stability in each condition with an eye tracker (see section on Fixation Stability Test in Method). We calculated the BCEA (bivariate normal ellipse area) values considering the fixation variability in the vertical and the horizontal directions. We found that neither the BCEA values among the pedestal, the annulus, and the disk adapter conditions differ (Figure 4 left panel) nor did they vary with the self-reported filling-in percept (Figure 4 right panel), even when the orientation shifts in different conditions were significantly different in both cases. Such results suggested that the TAE magnitude changes observed in experiments 1 and 2 cannot be explained by differences in fixation stability during adaptation.

## Conclusions

In experiment 1, we used three different adapters that occupied the center, the surround, and both the center and surround regions to induce TAE, which resulted in a perceived orientation shift of the target Gabor viewed in eccentric vision. Regardless of the adapter type, the orientation shift first increased then decreased as the adapter orientation moved further away from the target orientation, peaking between 10 degrees to 20 degrees, suggesting that the adaptation effect was orientation specific. The adaptation effect was strongest when the adapting stimulus had the same spatial extent as the target (pedestal condition, center-only). The adaptation effect decreased when the adapter surround was introduced (disk condition, center, and surround), indicating an inhibitory modulation from the surround to the center. This suggests that the surround induced lateral inhibition in the central region. The adaptation effect was smallest, but still significant, in the annulus (surround-only) condition when the adapter had no physical overlap with the target. In addition, observers who perceived stronger filling-in during the annulus adapter condition also perceived a stronger TAE.

In experiment 2, we used only the annulus adapter and recorded the subjectively reported filling-in percept after every trial to estimate the orientation shift separately for filling-in and non-filling-in trials. We discovered that the orientation shift was larger when filling-in occurred. Control measurements in a subgroup of participants indicated that our findings cannot be explained by differences in fixation stability across the different adaptation conditions. Together with the finding of a positive correlation between the strength of filling-in and the magnitude of the TAE in experiment 1, we conclude that the filling-in percept is associated with a stronger adaptation effect.

We adapted a divisive inhibition model to account for our results. In the model, we assumed that the observed adaptation effect was the result of an increase in the additive constant, *z*, in the denominator, leading to a reduction of the channel response after adaptation, as was observed in the literature (Foley & Chen, 1997). The magnitude of the adaptation effect is decided by the response of the local mechanism to the adapter, which is determined by the excitatory sensitivity parameter, *Se*, in the numerator. This sensitivity modulation has been shown to be crucial for surround modulation in other studies (Chen, Kasamatsu, Polat, & Norcia, 2001; Chen & Tyler, 2002).

## Supplementary Material

Supplement 1

Supplement 2

Supplement 3

Supplement 4

Supplement 5

Supplement 6
