# Supplementary material for: Lateral modulation of orientation perception in center-surround sinusoidal stimuli: Divisive inhibition in perceptual filling-in
Source: J Vis. 2020 Sep 4;20(9):5. doi: 10.1167/jov.20.9.5 (PMC7476660; doi:10.1167/jov.20.9.5)
Supplement: Supplement 2 [file jovi-20-9-5_s002.pdf]

Comparison between the PSI method and Psychometric function (PF) fitting results

Psychometric function (PF) fitting Procedure

We fitted a cumulative normal psychometric function (PF) to the raw data (the CCW trials) of each participant and examined the relationship between the estimates of this PF fitting results and the ones from the PSI method (the data in Figure 2a). To increase the number of trials per orientation level, we pooled trials from all runs of the same adapter condition together, and binned the data into 0.5° orientation steps from 0° to 15°. We calculated the proportion of responding CCW at each orientation step. We fitted a cumulative normal PF to the CCW proportion using the Matlab-based Palamedes toolbox (http://www.palamedestoolbox.org/) PF fitting function using a maximum likelihood criterion (PAL_PFML_Fit.m). Four parameters were returned for each PF fit, namely, alpha (threshold), beta (slope parameter), gamma (guessing rate or lapses), and lambda (finger-error rate). Gamma and lambda were fixed at values of 0.5 and 0 respectively, while alpha and beta were set as free parameters. Thus, the alpha value represents the threshold level at 75% CCW response. We used the Palamedes toolbox bootstrapping goodness-of-fit function (PAL_PFML_GoodnessOfFit.m) to determine goodness-of-fit of each fitted PF. We used 1000 simulations for each fitting result. We ruled out the fitting results that failed to return valid alpha and beta value (e.g. due to noisy data), returning alpha value lower than -3° (negative value means CW-oriented), or having poor goodness-of-fit (with less than 5% of the simulations having larger deviance/transformed likelihood ratio than that from the data).

Comparing parameters between the PSI estimates and PF fitting results

We calculate the Pearson correlation coefficient of the PSI estimated parameters and the PF fitting parameters of each participant. Figure 1 below demonstrates the relationship between the threshold estimates from the PSI method and the PF fitting to the raw data. The alpha/threshold value returned by the PF fitting represents the orientation corresponding to a 75% CCW reporting rate while the PSI estimate was estimated at 86% CCW response. To make a fairer comparison, we take the orientation level producing an 86% rate on the fitted cumulative normal function as the PF fitting estimate. If the estimates of the two methods agree well, there should be a significant positive correlation between the parameters, and the data points should lie close to the diagonal line.

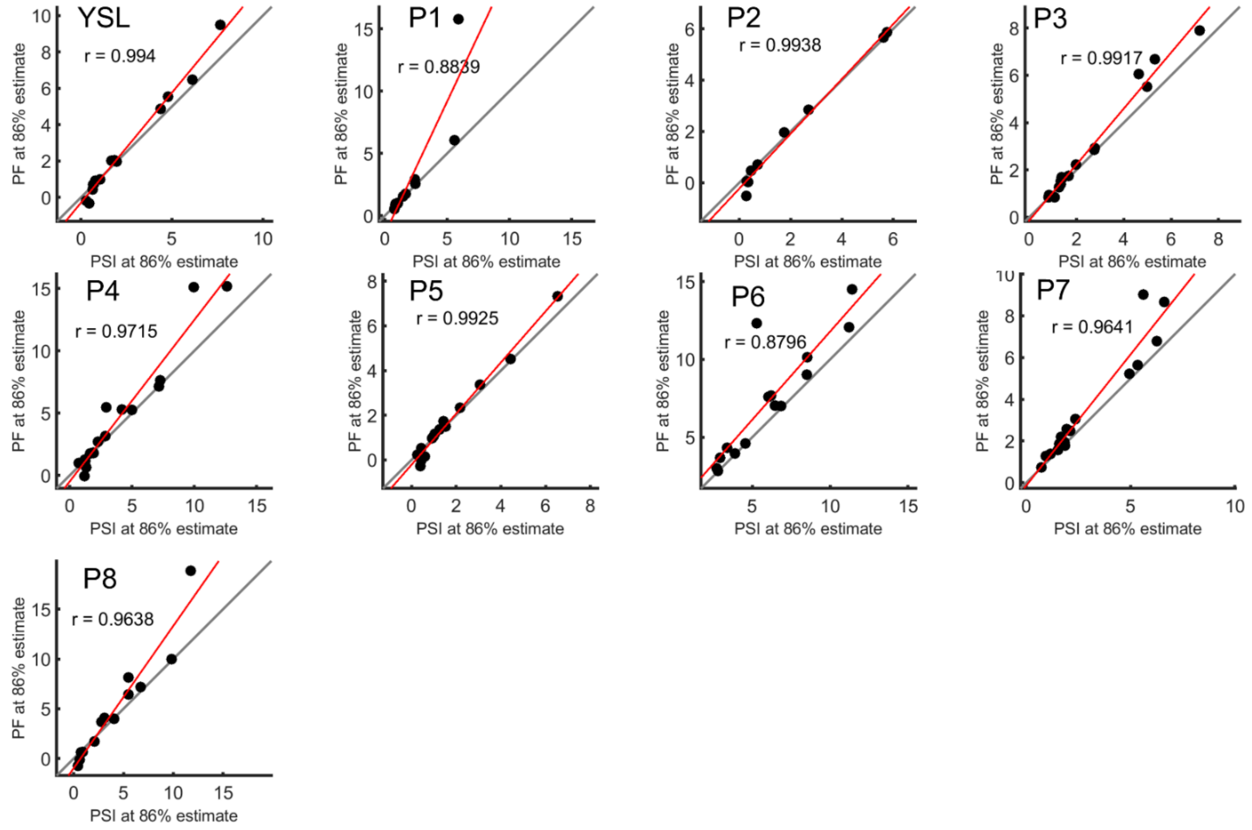

Figure 1. The correlation between the threshold estimates from the PSI method and the cumulative normal PF fitting results. The PSI estimate is plotted on the x-axis, while PF fitting on the y-axis. The solid red line represents the least-squares fitted regression line.

As can be seen in Figure 1, the threshold estimates from the two methods correspond reasonably well with each other, whereby the PF fitting estimate is higher than PSI estimate for data points of higher threshold value of some observers (i.e., the data points lie on the left side of the diagonal line). The correlation coefficient values and the *p*-values are shown in Table 1.

Table 1

Correlation between threshold estimates from PSI and PF method.

	LYS	*P1*	*P2*	*P3*	*P4*	*P5*	*P6*	*P7*	*P8*
PSI / PF threshold	.99	.88	.99	.99	.97	.99	.88	.96	.96
p-value	<0.001*	<0.001*	<0.001*	<0.001*	<0.001*	<0.001*	<0.001*	<0.001*	<0.001*
N	15	11	9	16	17	15	15	16	13

Notes. Each column represents values of one participant. The first row shows the correlation coefficient, while the second row the *p*-value between the threshold estimates of the two methods. The last row shows the number of data points. The * symbol indicates significant *p*-values ($p < 0.025$).

Relationship between the PF fitting threshold and slope

To examine the possible confound between the bias and unreliability (slope) in the data of each observer, we calculated the Pearson correlation coefficient between the 86% CCW responding rate and the slope value estimated from the PF fitting process. Figure 2 below shows such correlation, while Table 2 contains the correlation coefficient, the p-values as well as the number of data points for each participant (*N* differs due to the ruling out process mentioned in the PF fitting method). The data of two out of nine observers show a significant negative correlation between the slope and threshold values (*rs*(14) = -.51 and *rs*(14) = -.50, p = .021 and 0.024), while the data of the rest of the participants show no systematic relationship between the two estimates.

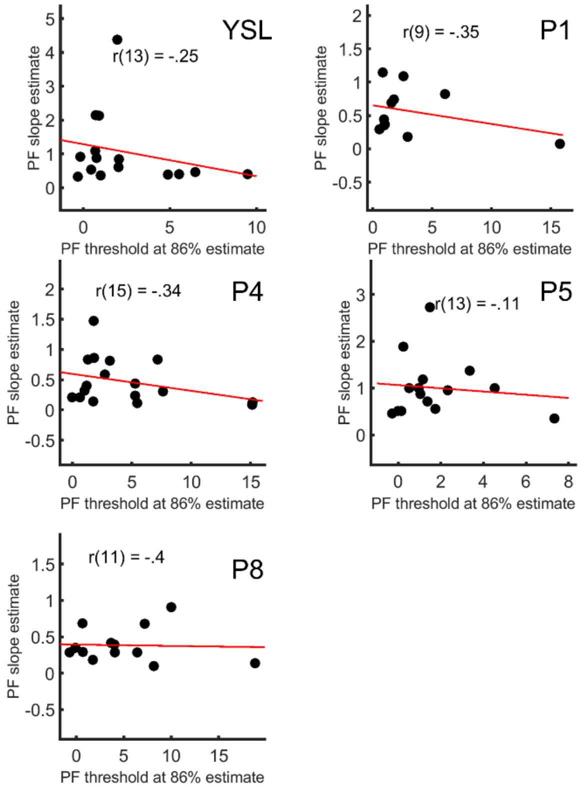

Figure 2. The correlation between the threshold and slope estimates PF fitting results. The threshold estimate is plotted on the x-axis, while slope on the y-axis. The solid red line represents the least-squares fitted regression line.

Table 2

Correlation between the threshold and slope estimates of the PF fitting results.

	LYS	*P1*	*P2*	*P3*	*P4*	*P5*	*P6*	*P7*	*P8*
Threshold/Slope	-.25	-.35	-.07	-.51	-.34	-.11	.24	-.50	-.04
p-value	0.186	0.146	0.424	0.021*	0.089	0.345	0.197	0.024*	0.449
N	15	11	9	16	17	15	15	16	13

Notes. Each column represents values of one participant. The first row shows the correlation coefficient, while the second row the *p*-value between the threshold estimates of the two methods. The last row shows the number of data points. The * symbol indicates significant *p*-values ($p < 0.025$).

Reanalysis of combined CW and CCW trial data using PF fitting process

Another way of examining the data in the current study is by combining the CW and CCW trials together and by fitting PF functions to the combined data set. To do so, we took the raw trial data (provided in Supplementary File S3) of each participant of the three oriented-adapter conditions (pedestal, annulus and disk adapter with five adapter orientation levels) in Experiment 1. We pooled all trials of the same adapter condition across different runs together. The CW trials were assigned with negative orientation values and we calculated the proportion of CCW response for each target (both CW and CCW) orientation. Then we again used the Palamedes toolbox function (PAL_PFML_Fit.m) to fit a cumulative normal Gaussian PF to each adapter condition. Alpha (threshold) and beta (slope) were free parameters while gamma (guessing rate) and lambda (finger error rate) were set as fixed parameter with the value 0.01. Thus, the threshold represents about 50% CCW response rate, i.e. the subjective verticality of the test stimulus in each condition.

Figure 3 below shows the averaged PF fitting parameters of all observers. The data are color-coded in the same manner as the Figure 2 in the main manuscript. The left panel shows the bias (alpha), while the right panel the JND (the inverse of slope/beta value). The bias estimate is very similar to the result of orientation shift in Figure 2a of the main manuscript, except that the bias values are lower. This was well expected since in the original PSI method, we estimated the 86% CCW rate. The JNDs of the pedestal condition (especially the 0 to 22.5°) are higher than the other two adapter type conditions. We suspected that the higher variability of data (larger

standard error) in the pedestal condition of most participants leads to such results. Such variability is captured by JND, which represents the unreliability in the data.

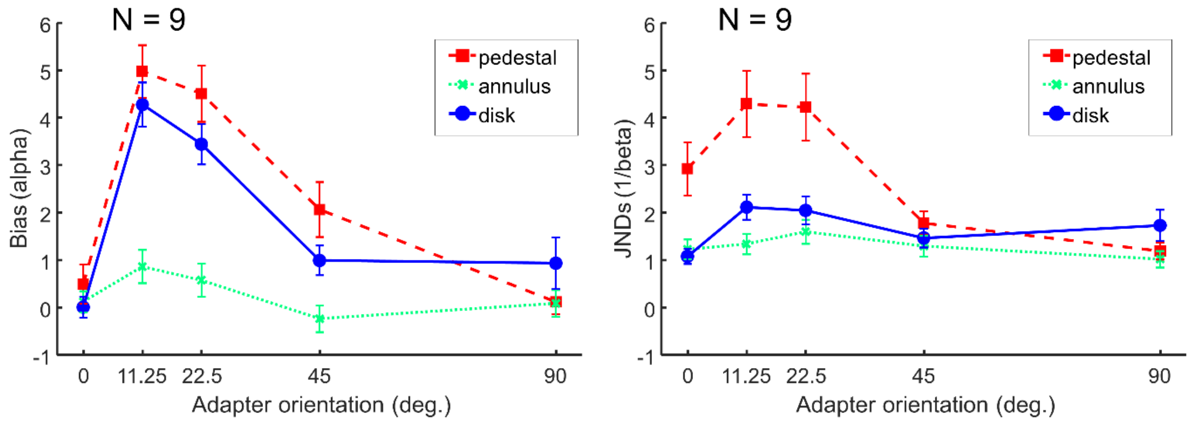

Figure 3. The averaged PF fitting results of combined CW and CCW trials of nine participants in the main experiment. In the left panel, the bias (alpha) is plotted against the adapter orientation. In the right panel, the JND is plotted against the adapter orientation. The error bars correspond to ±1 standard error of the mean.
