# Supplementary material for: Lateral modulation of orientation perception in center-surround sinusoidal stimuli: Divisive inhibition in perceptual filling-in
Source: J Vis. 2020 Sep 4;20(9):5. doi: 10.1167/jov.20.9.5 (PMC7476660; doi:10.1167/jov.20.9.5)
Supplement: Supplement 5 [file jovi-20-9-5_s005.pdf]

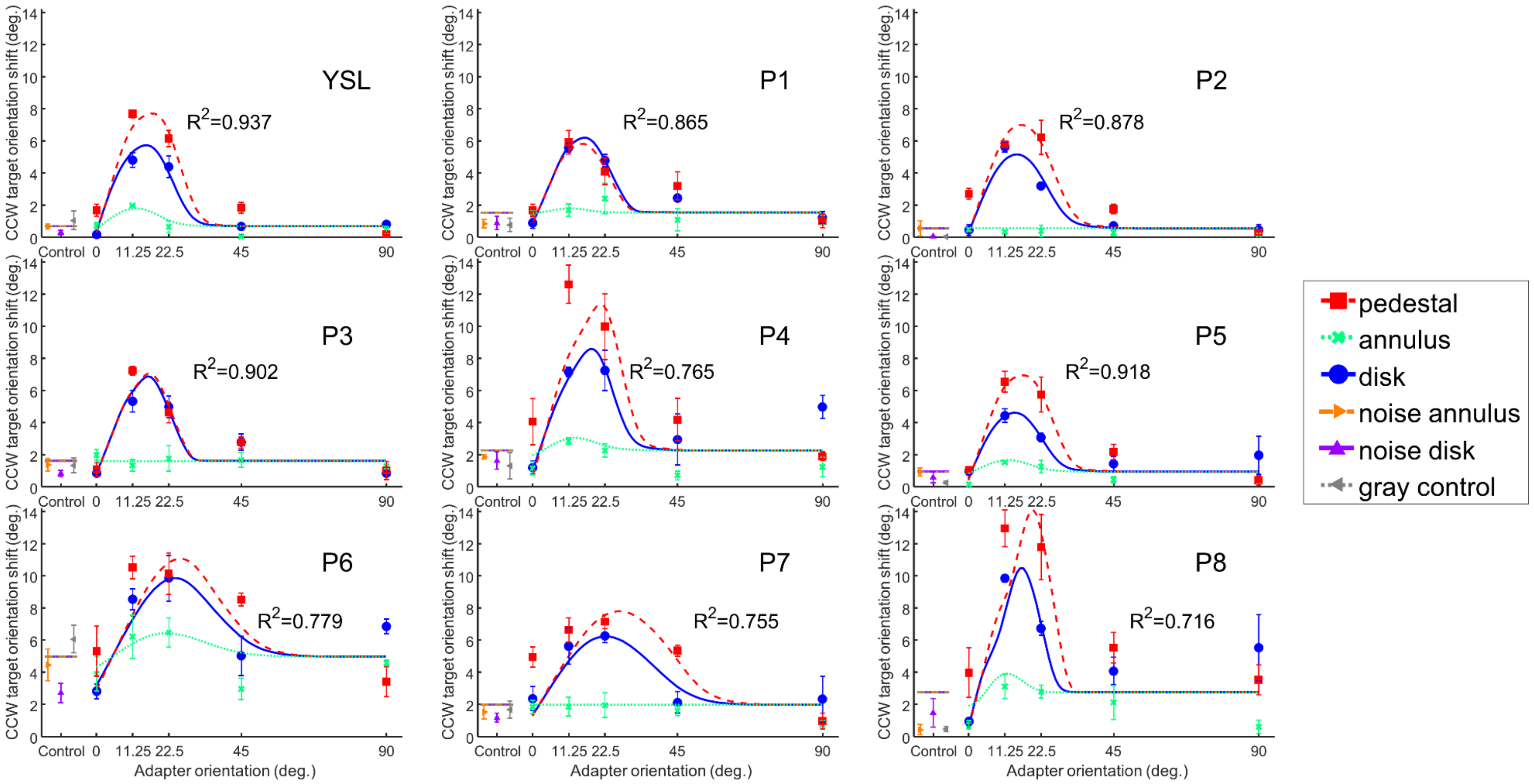

Figure S1. The individual data of the estimated CCW orientation shifts in Experiment 1. The symbols represent the behavioral data (red square: pedestal adapter; green cross: annulus adapter; blue circle: disk adapter, orange right-pointing triangle: noise annulus adapter; purple up-pointing triangle: noise disk adapter; gray left-pointing triangle: gray control/no adapter condition), while smooth curves and the horizontal lines the best fits of our computational model. The error bars are ±1 standard error of mean. See caption in Figure 2 in the main manuscript for further details.
