# Supplementary material for: Lateral modulation of orientation perception in center-surround sinusoidal stimuli: Divisive inhibition in perceptual filling-in
Source: J Vis. 2020 Sep 4;20(9):5. doi: 10.1167/jov.20.9.5 (PMC7476660; doi:10.1167/jov.20.9.5)
Supplement: Supplement 6 [file jovi-20-9-5_s006.pdf]

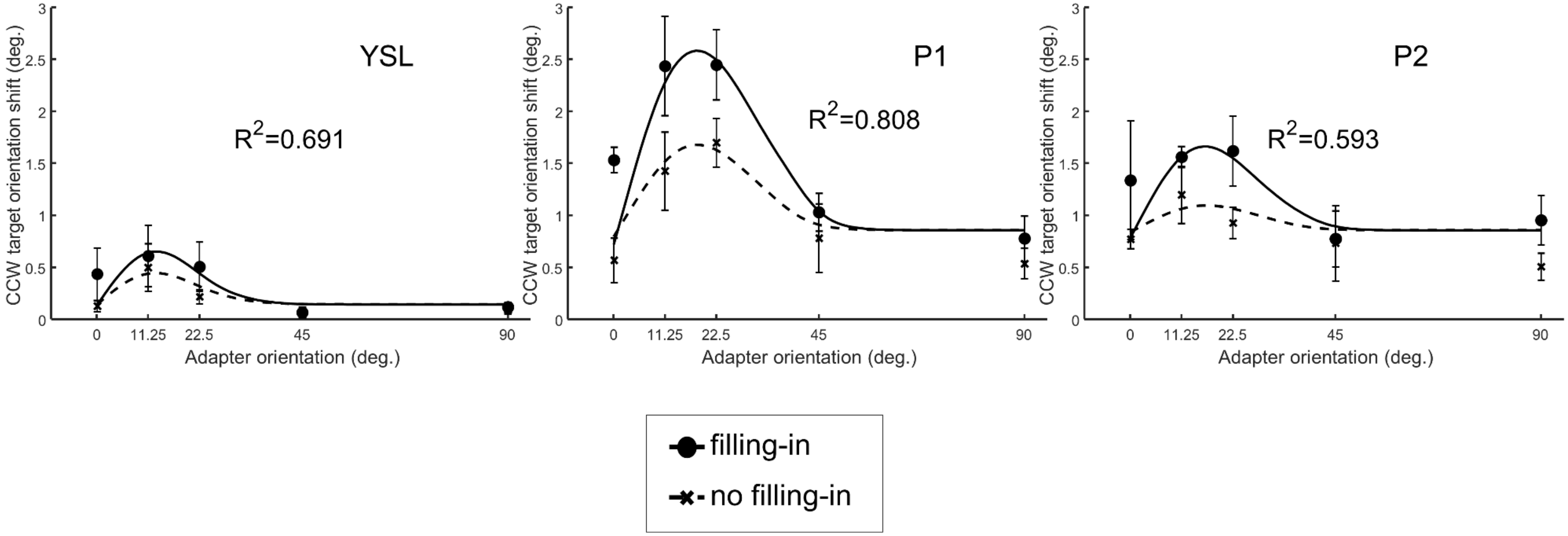

Figure S2. The individual data of the estimated CCW orientation shifts in Experiment 2. The symbols represent the empirical data (disk: with filling-in; cross: without filling-in), while smooth curves (solid curve: with filling-in; dotted curve: without filling-in) the best fits of our computational model. The error bars are ±1 standard error of mean. See caption in Figure 3 in the main manuscript for further details.
